# Re-evaluating frontopolar and temporoparietal contributions to detection and discrimination confidence

**DOI:** 10.1098/rsos.221091

**Published:** 2023-04-19

**Authors:** Matan Mazor, Chudi Gong, Stephen M. Fleming

**Affiliations:** ^1^ School of Psychological Sciences, Birkbeck, University of London, London WC1E 7HX, UK; ^2^ Wellcome Centre for Human Neuroimaging, University College London, London WC1E 6BT, UK; ^3^ Division of Psychology and Language Science, University College London, London WC1E 6BT, UK; ^4^ Department of Experimental Psychology, University College London, London WC1E 6BT, UK; ^5^ State Key Laboratory of Cognitive Neuroscience and Learning and IDG/McGovern Institute for Brain Research, Beijing Normal University, Beijing 100875, People's Republic of China; ^6^ Max Planck UCL Centre for Computational Psychiatry and Ageing Research, London WC1B 5EH, UK

**Keywords:** metacognition, detection, confidence, signal detection

## Abstract

Previously, we identified a subset of regions where the relation between decision confidence and univariate functional magnetic resonance imaging (fMRI) activity was quadratic, with stronger activation for both high and low compared with intermediate levels of confidence. We further showed that, in a subset of these regions, this quadratic modulation appeared only for confidence in detection decisions about the presence or absence of a stimulus, and not for confidence in discrimination decisions about stimulus identity (Mazor *et al.* 2021). Here, in a pre-registered follow-up experiment, we sought to replicate our original findings and identify the origins of putative detection-specific confidence signals by introducing a novel asymmetric-discrimination condition. The new condition required discriminating two alternatives but was engineered such that the distribution of perceptual evidence was asymmetric, just as in yes/no detection. We successfully replicated the quadratic modulation of subjective confidence in prefrontal, parietal and temporal cortices. However, in contrast with our original report, this quadratic effect was similar in detection and discrimination responses, but stronger in the novel asymmetric-discrimination condition. We interpret our findings as weighing against the detection-specificity of confidence signatures and speculate about possible alternative origins of a quadratic modulation of decision confidence.

## 1. Introduction

Adult humans are able not only to evaluate what they see and do not see, but also how confident they are in these percepts [1]. Investigations into the neural basis of metacognition reveal a network of brain regions where activation scales with perceptual confidence (for a coordinate-based meta-analysis, see [2]). However, a majority of previous computational modelling and neuroimaging studies of perceptual confidence have focused on understanding confidence in discrimination decisions (e.g. *was it a bird or a plane?*). By contrast, the computations and neural substrates supporting perceptual confidence for detection decisions (e.g. *was there anything there at all?*) remain largely uncharted territory. Mapping that territory is of considerable interest, both due to the conceptual overlap between (detection) confidence and perceptual awareness, and also because detection may invoke distinct computational demands that are not required in discrimination [3–5].

In a previous study [6], we compared the parametric effect of subjective decision confidence on brain activation in two perceptual decision-making tasks: a discrimination task (*was the grating tilted clockwise or anticlockwise?*) and a detection task (*was there any grating present at all?*). Replicating previous findings [2,7,8], we observed a linear effect of confidence in a set of predefined regions of interest, with high confidence levels associated with stronger (ventromedial prefrontal cortex, vmPFC; precuneus; ventral striatum) or weaker (posterior medial frontal cortex, pMFC) signals, across both tasks and responses. Exploratory analysis additionally revealed a widespread positive quadratic effect of confidence, with stronger signals associated with using the extreme ends of the confidence scale. In the right frontopolar cortex, right superior temporal sulcus (STS) and right pre-supplementary motor area (pre-SMA), this quadratic effect was stronger for the detection task, where participants decided whether a grating was present or absent. Additionally, in the right temporoparietal junction (rTPJ), a linear effect of confidence was stronger following judgements about target absence compared with judgements about target presence.

**Figure 1.**
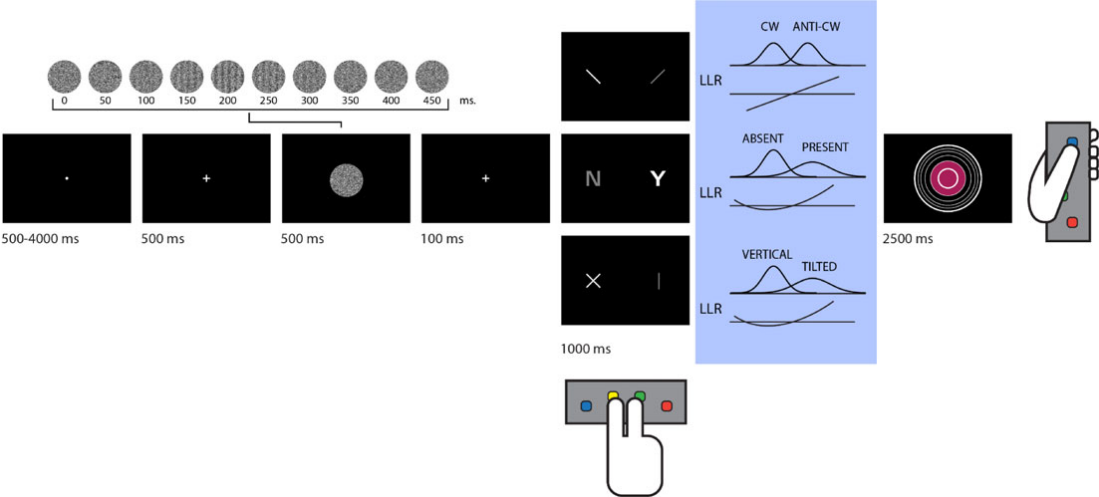
*Experimental design*. Stimuli consisted of dynamic random patterns of greyscale values. In all trials except for detection ‘target absent’ trials, a grating emerged from and disappeared back into the noise. Participants used the index and middle fingers of their right hand to indicate whether a grating was tilted clockwise or anticlockwise (discrimination), whether it was present or absent (detection), or whether it was vertical or tilted (tilt recognition). They then reported their level of confidence on a six-point scale by controlling the size of a coloured circle with their left thumb. In blue: inter-trial variability was similar for the two stimulus categories in discrimination, whereas in detection and tilt recognition, it was higher for one category over the other. LLR is a linear function of the perceptual sample in discrimination, but this relation is quadratic in detection and tilt recognition.

Signal detection-based computational simulations suggested that a quadratic activation profile may reflect the unequal variance nature of detection tasks (see figure 1). In detection, the variance associated with perceiving a signal is higher than the variance associated with perceiving the absence of a signal [9,10]. This unequal variance evidence structure can then produce a quadratic activation pattern in brain regions that are involved in dynamically updating a decision criterion or in representing the likelihood ratio between the two stimulus classes [6]. An alternative interpretation of our previous results is that distinct metacognitive processes are selectively invoked for decisions and confidence formation about presence and absence, but not in confidence about stimulus identity. For example, brain regions that selectively encode stimulus visibility [11], ones that correspond to higher-order nodes in a hierarchical model of perceptual states [5], or ones that are implicated in counterfactual thinking and attention monitoring [12] may show differential modulation of confidence in detection and discrimination decisions.

The design of our previous study did not allow us to decide between these alternative accounts. Here, we introduce a third hybrid condition to our experimental design: a discrimination task with the distributional properties of a detection task (*tilt recognition*; following [13]). This task requires subjects to report whether a grating is tilted or vertical: a discrimination judgement between two stimulus classes. However, because tilted gratings can appear in various orientations while vertical gratings are fixed, the distribution of perceptual evidence is of higher variance in the former, mimicking the variance asymmetry of a (yes/no) detection task. The two possible explanations of our previous findings (sensitivity of confidence encoding to variance structure versus a specific representation of presence and absence) thus make different predictions for this third condition. An unequal variance account predicts qualitatively similar neural confidence effects to those observed in detection, as the two tasks share a similar distributional structure. Conversely, a presence–absence asymmetry account predicts confidence effects that are qualitatively similar to those observed in discrimination, as the tilt-recognition task no longer requires inference about stimulus presence versus absence.

To anticipate our results, behavioural analysis confirmed that the tilt-recognition task induced detection-like unequal variance effects on subjects' confidence ratings—confirming that it created an asymmetric-discrimination task, as intended. A mass-univariate analysis of functional magnetic resonance imaging (fMRI) data replicated linear and quadratic effects of confidence in pre-specified regions of interest. However, unlike in our previous study, here the quadratic effects of confidence were similar in the detection and discrimination tasks and were instead stronger in the novel asymmetric-discrimination condition, which was also the condition with the most pronounced behavioural signatures of unequal variance. Furthermore, and in contrast with what we observed in Mazor *et al*. [6], in the rTPJ, a negative linear modulation of decision confidence was similar in detection ‘yes’ and ‘no’ responses. Representational similarity analysis (RSA) indicated that differences in multivariate activity patterns between high- and low-confidence trials were mostly task-invariant. We conclude with a discussion of how our previous conclusions should be revised in light of these new results.

## 2. Results

In a pre-registered design (pre-registered protocol folder: github.com/matanmazor/unequalVarianceDiscrimination/tree/main/experiment/protocolFolder/protocolFolder), a total of 46 participants performed three perceptual decision-making tasks while being scanned in a 3T MRI scanner: an orientation discrimination task (was the grating tilted clockwise or anticlockwise?), a detection task (was any grating presented at all?) and a tilt-recognition task (was the grating tilted or vertical?; see figure 1). Tasks were performed in separate blocks each comprising 26 trials. At the end of each trial, participants rated their confidence in the accuracy of their decision on a six-point scale. We adjusted the difficulty of the three tasks in a preceding behavioural session to achieve similar performance of around 70% accuracy. Fifteen to eighteen blocks were presented in five–six scanner runs.

### 2.1. Behavioural results

**Figure 2.**
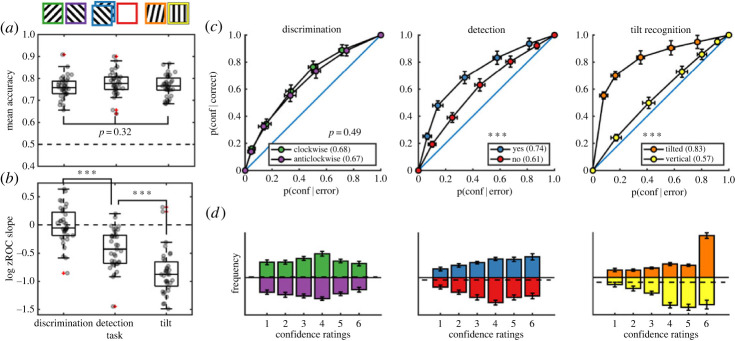
*Behavioural results*. (*a*) response accuracy was similar for the three tasks. (*b*) The log zROC slope was not different from 0 in discrimination, indicating similar variability in the representation of clockwise and anticlockwise stimuli. In detection, this quantity was significantly negative, indicating higher variability in the representation of signal. In tilt recognition, this quantity was even more negative, indicating higher variability in the representation of tilted stimuli. (*c*) Metacognitive sensitivity, quantified as the area under the response-conditional type-II ROC curve, was significantly higher for both ‘yes’ and ‘tilted’ responses compared with ‘no’ and ‘vertical’ responses, respectively. We observed no significant difference in metacognitive sensitivity between discrimination ‘clockwise’ and ‘anticlockwise’ responses. (*d*) Distributions of confidence ratings (on a 1–6 scale) for the three tasks and six responses. *** *p* < 0.001.

Thirty-five participants met our pre-registered inclusion criteria (see Methods). Task performance was similar for discrimination (76% accuracy), detection (78% accuracy) and tilt recognition (77% accuracy). Repeated measures analysis of variance revealed no difference in response accuracy between the three tasks (*F*_2,68_ = 1.17, *p* = 0.32, *BF*_01_ = 3.43; figure 2*a*). The probability of responding ‘clockwise’ in the discrimination task was 51% and not significantly different from 0.5 (*t*_34_ = 0.58, *p* = 0.57, *d* = 0.10). By contrast, participants were more likely to respond ‘no’ than ‘yes’ in the detection task (54% of all responses, *t*_34_ = 3.70, *p* < 0.001, *d* = 0.63) and ‘vertical’ than ‘tilted’ in the tilt-recognition task (57% of all responses, *t*_34_ = 6.67, *p* < 0.001, *d* = 1.13). This is consistent with an optimal setting of a decision criterion in an unequal variance setting [14].

Similar to what we had observed in Mazor *et al.* [6], participants were equally confident in reporting both clockwise (mean confidence on a 1–6 scale: *3.51*) and anticlockwise tilt (mean confidence: 3.44) in the discrimination task (*t*_34_ = 0.80, *p* = 0.43, *d* = 0.14). However, unlike in our previous study, here a numerical difference in mean confidence between detection ‘yes’ (3.95) and ‘no’ (3.80) responses was not significant (*t*_34_ = 1.62, *p* = 0.12, *d* = 0.27). Finally, in the tilt-recognition task participants were significantly more confident in reporting a tilted grating (4.60) than a vertical grating (4.19; *t*_34_ = 3.71, *p* < 0.001, *d* = 0.63; figure 2*d*).

Confidence ratings are typically aligned with objective accuracy, such that participants are more confident on average when they are correct, compared with when they are wrong. Previous studies found that this alignment, commonly referred to as *metacognitive sensitivity*, is reduced for decisions about target absence compared with decisions about target presence [6,10,15,16]. Here also, metacognitive sensitivity (quantified as the area under the response-conditional type-II receiver operating characteristic (ROC) curve) was significantly higher for detection ‘yes’ compared with ‘no’ responses (*t*_34_ = 6.41, *p* < 0.001, *d* = 1.1; figure 2*c*). Similarly, in the tilt-recognition task, metacognitive sensitivity was higher for ‘tilted’ compared with ‘vertical’ responses (*t*_34_ = 9.55, *p* < 0.001, *d* = 1.61). No difference in metacognitive sensitivity was observed between discrimination clockwise and anticlockwise responses (*t*_34_ = 0.70, *p* = 0.49, *d* = 0.12).

In an unequal variance signal detection setting, metacognitive sensitivity is expected to be higher for classifying a stimulus as belonging to the high-variance compared with the low-variance stimulus class. Our tilt-recognition task is an example of such a setting: the ‘vertical’ class had low variance (all stimuli were vertical), and the ‘tilted’ class had high variance (some stimuli were more tilted than others). As expected, the ratio between the standard deviations of the two stimulus categories (measured as the geometric mean of type-1 zROC slopes), was 0.55 and significantly lower than 1, indicating higher variability in the representation of tilted stimuli (a *t*-test performed on log-slopes against 0: *t*_34_ = −12.50, *p* < 0.001, *d* = 2.11; figure 2*b*). Similarly, this ratio was 0.74 for the detection task, indicating higher variability in the encoding of target presence (*t*_34_ = −7.27, *p* < 0.001, *d* = 1.23). By contrast, the ratio was 0.99 for the discrimination task and statistically indistinguishable from 1, indicating similar variability in the encoding of clockwise and anticlockwise stimuli (*t*_34_ = −0.26, *p* = 0.79, *d* = 0.04).

### 2.2. Imaging results

Whole-brain results for all pre-registered contrasts are available on NeuroVault (https://identifiers.org/neurovault.collection:12352). Anonymized imaging data from all included subjects are available on OpenNeuro (https://openneuro.org/datasets/ds004081/versions/1.0.0). All analyses were run in Matlab R2022. Analysis scripts are available at https://github.com/matanmazor/unequalVarianceDiscrimination.

We pre-registered a plan to evaluate the parametric modulation of confidence both directly from brain activations, as well as indirectly from beta coefficients of a design matrix where confidence is specified as a categorical variable. This two-step solution controls for metacognitive biases in that all confidence levels equally contribute to parametric modulation estimates, regardless of their frequency in the data. Results from the categorical design matrices mostly agreed with those from the parametric modulation analysis. We therefore report the parametric modulation results and mention when the categorical design matrix provided conflicting results. Full results from both approaches are presented in the electronic supplementary material.

#### 2.2.1. Linear and quadratic effects of confidence

Our primary (quadratic confidence) design matrix included parametric modulators for linear and quadratic effects of confidence. Among our pre-specified regions of interest, the medial frontopolar (FPm) and vmPFC regions of interest (ROIs) showed a positive linear effect of confidence (FPm: *t*_34_ = 3.45, *p* < 0.001, *d* = 0.58, vmPFC: *t*_34_ = 4.53, *p* < 0.001, *d* = 0.77). A whole-brain contrast revealed a positive modulation of confidence in the bilateral precuneus, claustrum and ventral striatum. Conversely, the rTPJ showed a negative linear modulation of confidence, similar to what we observed in our previous study (*t*_34_ = −3.35, *p* < 0.001, *d* = 0.57). Quadratic polynomials fitted to beta values from the categorical design matrices revealed a negative linear modulation of confidence also in the right STS (*t*_34_ = 2.47, *p* < 0.05, *d* = 0.42), and a whole-brain analysis revealed a negative linear effect of confidence in the pMFC.

Consistent with what we had observed in our previous study, a positive quadratic effect of confidence was robustly observed in a number of regions. Among our pre-specified ROIs, this effect was significant in lateral frontopolar cortex (FPl; *t*_34_ = 2.93, *p* < 0.01, *d* = 0.50), Brodmann area 46 (BA46; *t*_34_ = 4.43, *p* < 0.001, *d* = 0.75), rTPJ (*t*_34_ = 4.89, *p* < 0.001, *d* = 0.83), right STS (rSTS) (*t*_34_ = 3.62, *p* < 0 .001, *d* = 0.61) and pre-supplementary motor area (pre-SMA; *t*_34_ = 5.00, *p* < 0.001, *d* = 0.85). Whole-brain analysis revealed a quadratic effect of confidence also in dorsolateral prefrontal and orbitofrontal cortex, anterior insula, precuneus, posterior cingulate and in the cerebellum. Similar effects were observed when directly controlling for motor aspects of the confidence-rating phase (see electronic supplementary material, S18).

#### 2.2.2. Task-specific activations

We next asked whether brain activation differed between the three tasks, collapsed across responses and confidence levels. Repeated measures analyses of variance failed to find a main effect of task in any of our seven pre-registered ROIs (all *p*s > 0.33; see electronic supplementary material, S3). Outside these regions, whole-brain analysis (*p* < 0.05, corrected for family-wise error at the cluster level) revealed that activations in bilateral premotor cortex were sensitive to task identity. This is consistent with the successful encoding of semantic meaning of motor actions from associative motor cortex [17].

#### 2.2.3. Task- and response-specific confidence modulations

**Figure 3.**
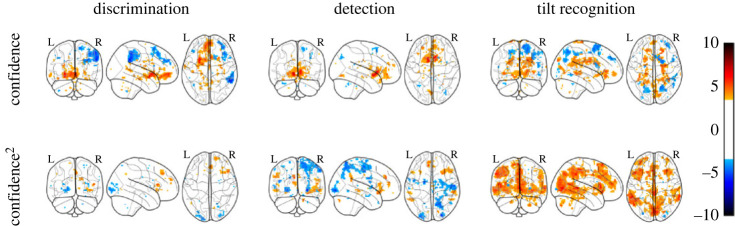
Linear (first row) and quadratic (second row) effects of confidence for the three tasks. *p* < 0.001, uncorrected for multiple comparison.

We next asked whether confidence-related brain activation differed between the three tasks. A linear modulation of confidence was similar for the three tasks: repeated measures ANOVAs revealed no effect of task in any of our ROIs (all *p*s > 0.30; see electronic supplementary material, S4 and figure 3, first row), and whole-brain analysis revealed no differences outside these pre-specified regions.

Our main hypothesis, however, was that a quadratic modulation of confidence should be more pronounced in some tasks than in others—indicating presence–absence or unequal variance-related asymmetries. Within our ROIs, this was the case in BA46 (*F*_2,34_ = 4.47, *p* < 0.05), rSTS (*F*_2,34_ = 3.80, *p* < 0.05) and marginally in rTPJ (*F*_2,34_ = 2.1, *p* = 0.08). The same regions showed a marginal effect when subjecting betas from the categorical design matrix to a group-level ANOVA (*p* = 0.06, *p* = 0.08 and *p* = 0.06 for BA46, rSTS and rTPJ, respectively). Whole-brain analysis further revealed differences in a quadratic modulation of confidence across tasks in the insula (*p* < 0.05, cluster-corrected).

Our next set of pre-registered tests was designed to pinpoint the origins of this interaction of the quadratic expansion of confidence with task. First, we attempted to replicate our finding from study 1 of a stronger quadratic modulation of confidence in detection compared with discrimination. Contrary to our prediction, we found no significant differences in modulation in any of our ROIs (all *p*s > 0.17, see electronic supplementary material, S6). To determine whether this absence of a significant result should also be taken as positive evidence against a difference between detection and discrimination, we subjected our data to a Bayesian *t*-test [18]. In the FPm ROI, we obtained moderate evidence *against* a difference between detection and discrimination (*BF*_01_ = 3.71). Similarly, we obtained moderate evidence against a difference between detection and discrimination in the pre-SMA (*BF*_01_ = 5.43). Analysing beta values from the categorical design matrix, we obtained moderate evidence for the null hypothesis of no difference also in FPl (*BF*_01_ = 3.04) and rTPJ (*BF*_01_ = 5.48). Bayes factors for all other ROIs in which we observed an effect in study 1 were within the interval [⅓,3], indicating no clear evidence for or against an effect (see electronic supplementary material, S6).

In contrast with detection, where quadratic confidence profiles were similar to discrimination, the quadratic effect of confidence was significantly stronger in the tilt recognition relative to the discrimination task in BA46 (*t*_34_ = 2.23, *p* < 0.05), rTPJ (*t*_34_ = 2.18, *p* < 0.05), rSTS (*t*_34_ = 2.86, *p* < 0.01) and marginally in pre-SMA (*t*_34_ = 1.78, *p* = 0.08).

In our original study [6], a cluster in the rTPJ showed a negative linear effect of confidence, which was significantly more negative in detection ‘no’ compared with ‘yes’ responses. In the current study, activation in this region again showed a negative linear, as well as a positive quadratic effect of confidence. However, an interaction between confidence and detection response was not significant (*t*_34_ = 0.72, *p* = 0.47). A Bayesian *t*-test provided moderate evidence against a difference between detection ‘yes’ and ‘no’ responses in this region (*BF*_01_ = 4.33). No other region of interest showed an interaction of confidence with detection response (all *p*s > 0.23, see electronic supplementary material, S8). Similarly, none of our ROIs showed a significant interaction of confidence with response in the tilt-recognition task (all *p*s > 0.11; see electronic supplementary material, S9). A whole-brain analysis revealed a cluster in the left lingual gyrus (MNI coordinates [−30, −64, −8]) in which a linear modulation of confidence was stronger in ‘tilt’ compared with ‘vertical’ responses (*p* < 0.05, cluster-corrected).

#### 2.2.4. Multivariate analysis

**Figure 4.**
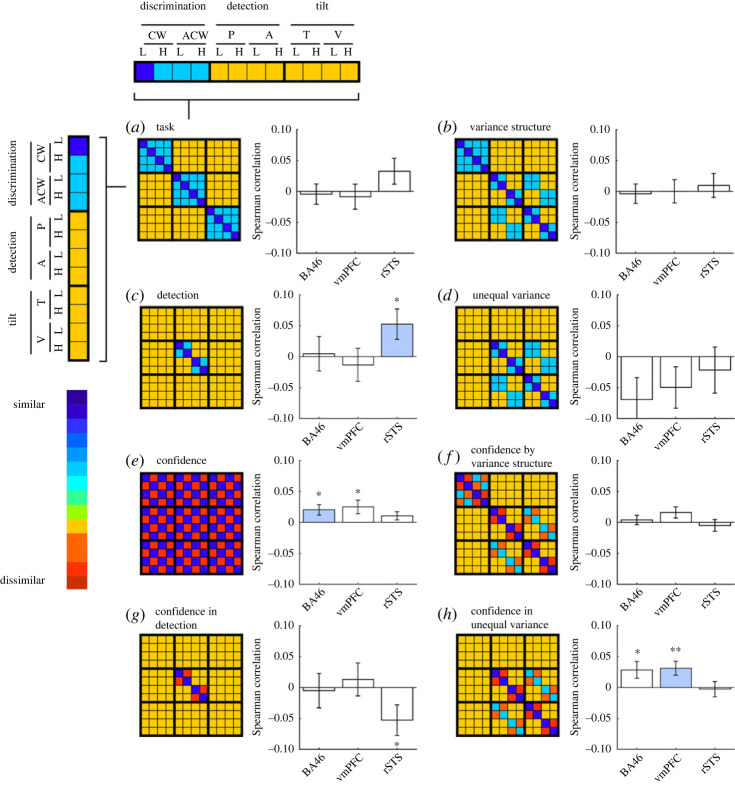
The eight *a priori* RDMs used for RSA, and their corresponding Spearman correlations with empirical RDMs from the three ROIs. For each ROI, we marked the RDM that produced the most robust correlation (in standardized effect sizes) in blue. *p < 0.05; **p < 0.01.

To further investigate the relationships between spatial activation patterns across tasks, responses and confidence levels, we next turned to representational similarity analysis [19]. Specifically, we asked which regions represented task, irrespective of confidence; which represented confidence, irrespective of task; and which represented confidence in a task-dependent manner. We pre-registered eight representational dissimilarity matrices (RDMs), each specifying a theory-based prediction regarding which trials should be encoded similarly or dissimilarly based on task, response and reported confidence (figure 4) and compared them against empirical dissimilarity matrices extracted from our pre-registered ROIs.

First, we used the median confidence rating for each response to separate high- and low-confidence responses (for a similar analysis using a response-invariant confidence cut-off with similar results, see electronic supplementary material, S16 and S17). We then computed the empirical similarity in spatial activation patterns between the 12 trial categories (3 tasks × 2 responses × high or low confidence). In order to verify that empirical RDMs hold reliable condition-specific multivariate activation patterns, we compared on- and off-diagonal entries in the ranked RDMs. If condition-specific information is encoded in RDMs, off-diagonal distances should be higher than on-diagonal ones [20]. This was the case in FPm (*t*_34_ = 3.29, *p* < 0.01), BA46 (*t*_34_ = 2.87, *p* < 0.01), vmPFC (*t*_34_ = 2.06, *p* < 0.05) and rSTS (*t*_34_ = 2.17, *p* < 0.05), but not in FPl, rTPJ and pre-SMA (all *p*s > 0.17). In the FPm ROI, subject-specific RDMs were not predicted by the averaged RDM of other subjects (*p* = 0.38), reflecting poor multivariate signal in this region [21]. We therefore restricted the multivariate analysis to the three regions with reliable multivariate activation patterns: BA46, vmPFC and rSTS. In all following analyses, on-diagonal entries are ignored, in order not to artificially inflate correlation measures [20].

In the rSTS ROI, multivariate activation patterns were most consistent with encoding of detection responses (target present or absent; RDM C, figure 4*c*; *p* < 0.05). A negative correlation with RDM G (detection confidence) is driven by the perfect negative correlation between RDMs C and G, when excluding the diagonal entries.

By contrast, in the prefrontal BA46 and vmPFC ROIs, multivariate brain activation patterns were most consistent with task-invariant confidence encoding (RDM E, figure 4*e*; *p*s < 0.05), and with confidence encoding that is specific to an unequal variance setting (RDM H, figure 4*h*, *p*s < 0.05). Spearman correlations with RDMs E and H were not significantly different from each other (*p*s > 0.54). This is in line with results from our previous study [6], where cross-classification analysis revealed no evidence for task-specificity in multivariate confidence representations (see full pre-registered analyses https://osf.io/y3ftk/, section ‘Task-specific and task-invariant confidence representation’).

**Figure 5.**
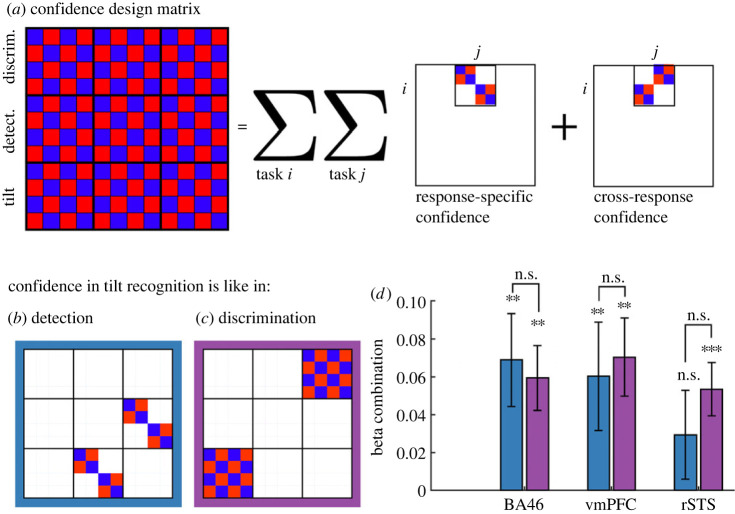
Multiple regression analysis. RDM E from figure 4 was broken down into 18 constituent RDMs, which were then used to predict empirical RDMs in the seven ROIs (*a*). We then used beta coefficients from this multiple regression analysis to produce two beta combinations encoding our two hypotheses about differences between tasks (*b*,*c*): the first corresponds to similarity in confidence encoding between the tilt-recognition and the detection tasks, and the second to similarity in confidence encoding between the tilt-recognition and discrimination tasks. We found no significant differences between these two weighted contrasts (*d*), supporting a task-invariant account of confidence encoding in these regions. **p < 0.01; ***p < 0.001; n.s. *p* > 0.05.

To further explore the sensitivity of confidence encoding to variance structure, we subjected the empirical RDMs to a multiple regression analysis in which candidate RDMs competed to explain the variance in the empirical RDM. First, we decomposed RDM E into 18 constituent sub-RDMs. Each such RDM represented the similarity between confidence encoding in two tasks, or within a single task (figure 5*a*). We focused on specific sub-RDMs for which a variance structure account made unique predictions. In particular, a variance structure account predicts a response-specific similarity between confidence in detection and in tilt recognition (figure 5*b*), but a presence–absence account predicts a similar response-invariant encoding of confidence in tilt recognition and in discrimination (figure 5*c*). To test this prediction, we compared the weighted coefficient combinations for these two predictions.

In the two prefrontal regions, vmPFC and BA46, multivariate activation patterns in the tilt-recognition task were similar both to multivariate activation patterns in the discrimination task (vmPFC: *t*_34_ = 3.41, *p* < 0.01; BA46: *t*_34_ = 3.48, *p* = 0.001), and to multivariate activation patterns in the detection task (vmPFC: *t*_34_ = 2.10, *p* < 0.05; BA46: *t*_34_ = 2.81, *p* < 0.01). Consistent with our RSA results, this finding is in line with a task-invariant multivariate representation of confidence in these regions.

By contrast, rSTS showed similar confidence encoding in the tilt-recognition and discrimination tasks (*t*_34_ = 3.79, *p* < 0.001), but distinct confidence encodings in tilt-recognition and detection tasks (*t*_34_ = 1.25, *p* = 0.22). Still, we observed no significant difference between these correlations (*t*_34_ = 0.84, *p* = 0.41; figure 5*d*), providing only indirect support for the hypothesis that multivariate confidence encoding in rSTS was different for detection and discrimination.

## 3. Discussion

In a previous study we identified distinct neural contributions to confidence in perceptual detection that were not observed in a performance-matched discrimination task. In this pre-registered follow-up study, we set out to replicate this finding and to further characterize the computational origins of a quadratic modulation of confidence. In what follows, we summarize our findings, discuss how our previous conclusions should be revised in light of this new data and unpack what this may mean for our understanding of a quadratic effect of decision confidence in association cortex.

### 3.1. A quadratic modulation of confidence is not distinct to perceptual detection

As in Mazor *et al*. [6], here too we observed a widespread quadratic modulation of subjective confidence in prefrontal and parietal association cortex. In our previous study, this effect was stronger in a perceptual detection task, and in some frontopolar regions was not observed at all in a performance-matched discrimination task. By contrast, in the present study this effect was no longer specific to detection, and was instead observed in both detection and discrimination tasks. When considered together, the results of both studies provide no clear evidence for or against the detection-specificity of quadratic confidence responses in FPl and FPm (*BF*_01_ = 2.60 and *BF*_01_ = 1.47, respectively, calculated as the product of the two Bayes factors from Exp. 1 and 2, using a scaling factor of 0.707 over effect sizes).

There are a number of potential reasons for this difference between our results here and those reported in our previous study; for example, in our previous study stimuli were presented briefly, whereas here we opted for a dynamic display mode (see Methods). Alternatively, the inclusion of a third task where difficulty is manipulated differently may have had an indirect effect on participants' disposition towards their confidence ratings in the detection and discrimination tasks. An alternative interpretation is that in our previous study a difference between detection and discrimination was a false positive driven by noise, which happened to facilitate the quadratic trend in one task and reduce it in another. Indeed, this difference in a quadratic modulation was not *a priori* expected based on theory in our previous study and was the result of exploratory analysis that went beyond our pre-registration. The current pre-registered, hypothesis-driven replication provides an unbiased test of this effect, which resulted in a failure to replicate it.

Surprisingly, however, the third ‘hybrid’ tilt-recognition task which we introduced—a discrimination task with the signal detection properties of a detection task—produced the strongest quadratic modulation of confidence in a number of regions of interest. Specifically, in BA46, rTPJ and rSTS, a quadratic modulation of confidence was significantly more pronounced in tilt recognition than in the discrimination task. Although this result is difficult to interpret without it being accompanied by a significant difference in quadratic modulation for detection (and thereby being consistent with a signature of unequal variance), it nevertheless provides some support that a quadratic modulation of confidence-related brain activity may be sensitive to the variance structure of perceptual evidence. Indeed, the hybrid tilt-recognition task showed exaggerated behavioural markers of unequal variance compared with both the detection and discrimination tasks (in both zROC curves and rcROCs; figure 2). Our results are therefore consistent with a graded sensitivity of this neural marker to variance structure. By contrast, the fact that the strongest quadratic modulation was observed in decisions about stimulus category (tilt versus vertical), rather than stimulus presence or absence, strongly suggests that this effect is not driven by a qualitative difference between detection and discrimination decisions.

### 3.2. A similar linear modulation of confidence for detection ‘yes’ and ‘no’ responses in right temporoparietal junction

In our previous study, whole-brain analysis revealed a cluster in the right posterior TPJ in which a linear modulation of decision confidence was more negative for detection ‘no’ compared with ‘yes’ responses. We interpreted this finding in light of a potential role for attention monitoring in inference about absence, where participants are required to differentiate failures to perceive the target due to target absence and due to lapses of attention, and in light of an involvement of the temporoparietal junction in modelling attention states [22].

In this second experiment, we pre-registered our plan to directly test this hypothesis, defining the rTPJ based on the relevant contrast in our first study. Contrary to our expectation, a negative modulation of decision confidence in the rTPJ ROI was similar for the two detection responses (see electronic supplementary material, S13). A Bayesian *t*-test provided moderate evidence for the null hypothesis that confidence encoding in this region is invariant to detection response.

### 3.3. Multivariate analysis provides weak evidence for distinct confidence encoding in an unequal variance setting

Using RSA, we compared multivariate activation patterns in our pre-specified ROIs against theory-driven representational similarity matrices (figure 4). This analysis revealed robust encoding of decision confidence in BA46 and vmPFC, consistent with a representation of decision confidence that is either task-invariant or sensitive to the variance structure of a task. By contrast, multivariate activation patterns in rSTS were most consistent with a representation of detection decisions about stimulus presence or absence. Follow-up multiple regression analysis revealed that rSTS similarly encoded decision confidence in the two discrimination tasks, but that these confidence representations were not shared with the detection task. This exploratory analysis provides some indirect support for different neuronal mechanisms underlying metacognitive evaluations of decisions about presence and absence, versus stimulus category.

### 3.4. Power considerations in neuroimaging research

We note that our target sample size (*N* = 35 included subjects) was based on an *a priori* power calculation to obtain 65–85% power to replicate our previous findings, assuming no inflation of effect sizes in the original report, and no effect of a reduction in the number of trials per task for group-level sensitivity. Our power and sample size aspirations were balanced with resource constraints (grant funding and time, given that *N* = 46 subjects were tested to obtain *N* = 35 included subjects), but remain substantially above recent estimates in fMRI research, where power can be routinely lower than 10% ([23]; though we note that power calculations for whole-brain analyses are complicated by the need to deal with mass-univariate multiple comparisons correction). Low statistical power hinders the field's ability to create a progressive research programme in which one set of findings builds on the other.

Even with medium-range power of approximately 75%, and assuming a true effect, we should expect mixed results in a series of studies (roughly 1 in 3 null results with an alpha level of 0.05 for individual tests). As mentioned above, our original finding of a difference in detection and discrimination was the result of exploratory analysis, probably heightening the probability of a type 1 error. Our suspicion is that our current study would, in a previous era, have languished unpublished—a casualty of the file drawer effect. We think it is critically important that such studies are now published to allow a full picture of the strength of different effects. More generally, our findings highlight the importance of allocating funding to replication studies in cognitive neuroscience and the importance of pre-registration of hypothesis tests. With these considerations in mind, we therefore turn in the remainder of the discussion to offer some interpretations of the current findings and highlight questions for future research.

### 3.5. A quadratic modulation of confidence: ideas and speculations

The previous sections summarize the current picture of our findings, in light of our pre-registered hypotheses, and describe both the similarities and differences between the current results and those of our previous paper. A particularly strong and consistent finding across both studies was that univariate fMRI activation in prefrontal and parietal cortex is quadratically modulated by decision confidence. However, as described above, we find no clear support for or against our hypothesized variance structure account of a quadratic modulation of decision confidence. This therefore leaves underdetermined the computational basis of such an effect. In the following, we discuss two additional candidate interpretations of a quadratic modulation of confidence.

In Mazor *et al*. [6], we referred to two previous reports of a quadratic relation between subjective ratings and brain activation: one in subjective visibility ratings [24] and the other in product desirability ratings [25]. We then explained that our findings were qualitatively different: while a quadratic effect for visibility or product desirability can reflect a linear modulation of subjective confidence when both ends of the rating scale are associated with higher levels of confidence (for example, being highly confident that a product is or is not desirable, or that a stimulus is or is not visible), our results highlight a quadratic effect in confidence itself. In other words, the low end of the scale should reflect low confidence in the perceptual decision, rather than high confidence in a negative rating.

However, although the low end of the confidence scale reflects low confidence *in a decision*, it may still reflect a high level of confidence *in the confidence rating itself*.^1^ With our incentive structure, reward is dependent not only on task performance but also on the adequacy of confidence reports (see Methods). It is therefore not unlikely a participant would reason something along the lines of ‘I'm not sure what I just saw, so I'm highly confident that I should rate my subjective confidence as low to maximize my bonus'. Brain regions where activation scales with subjective confidence may then reflect this meta-level subjective confidence (effectively, confidence in confidence) with a higher level of activity not only for the upper end, but also for the lower end of the confidence scale. Note this scheme does not imply the existence of ‘meta-meta-cognition’: all that is required is that confidence ratings are represented as part of the primary (type-1) task, such that metacognitive resources are available to evaluate the quality of confidence reports themselves [26].

It then remains to be explained why a quadratic effect of confidence is significantly stronger in the tilt-recognition compared with both discrimination and detection tasks (figure 3; electronic supplementary material, figure S7). One possibility is that confidence in the presence or absence of a tilt more naturally lends itself to rule-based heuristics (such as a mapping between perceived angle and confidence level), leaving metacognitive resources free to monitor the quality of subjective confidence ratings.

Alternatively, a quadratic modulation of decision confidence may reflect object-level (that is, not meta-level) inter-trial fluctuations in visual attention. As an example of how this might arise, a recent EEG study [27] revealed a negative linear relation between reported attention and pre-stimulus alpha power in the 8–12 Hz frequency band. By contrast, high levels of decision confidence were associated with intermediate pre-stimulus alpha power, giving rise to a negative quadratic relation between alpha power and confidence. One implication of this result is that brain regions where activation is typically negatively correlated with alpha power may show a positive quadratic modulation of confidence in virtue of their relation to pre-stimulus alpha. This potentially explains why a quadratic modulation of confidence is prominent in a frontoparietal network where activity has been negatively linked to alpha power [28,29].

It is unclear, however, why a putative relationship between neural correlates of attention and subjective confidence should be sensitive to the variance structure of the task. One useful approach is to ask how, under Bayesian decision theory, decisions and confidence estimates should adjust to reflect differences in the effects of attention on internal distributions. For instance, in a behavioural study [13], participants rated their confidence in whether the orientation of a grating was sampled from a wide or narrow distribution, both centred at 0 degrees. A comparison of trials with valid, invalid and neutral cues revealed that participants rationally adapted their decisions and confidence estimates to their current attention state. Note that this effect of attention on perceptual decisions is specific to unequal variance settings; in an equal variance setting, accuracy is highest when the decision criterion is set to midway between the two stimulus distributions, regardless of sensory precision. By contrast, an unequal variance setting introduces a link between optimal placement of the decision criterion and sensory precision. As we show in Mazor *et al*. [6], a model where subjects dynamically adjust their decision criterion based on previous samples displays different associations between criterion adjustment and confidence, depending on the variance structure of the task. Together, a possible interpretation of our findings is that a quadratic modulation of confidence in regions such as BA46, rTPJ and rSTS is effectively mediated by decisions about policy changes, such as adjustments of a decision criterion based on observed samples.

## 4. Conclusion

In conclusion, in a pre-registered experiment we find that a quadratic effect of decision confidence on brain activity is not specific to decisions about presence and absence, but may be sensitive to the variance structure of the task. We discuss three candidate accounts of this effect, one postulating a role for subjective confidence in the accuracy of confidence ratings themselves, one identifying this quadratic effect with the neural correlates of fluctuations in attention, and one linking brain activations to the online adjustment of a decision criterion.

## 5. Methods

We report how we determined our sample size, all data exclusions (if any), all manipulations and all measures in the study. All design and analysis details were pre-registered before data acquisition and time-locked using pre-RNG randomization: we used the SHA256 hash function to translate our pre-registered protocol folder (https://github.com/matanmazor/unequalVarianceDiscrimination/blob/main/experiment/protocolFolder.zip) to a series of bits (7c2c27da12b6768b1789907ba5d2ec46b45d302199d5368795879fcff844d043). These bits were then used to initialize Matlab's pseudorandom number generator for determining the order and timing of experimental events (relevant lines in the experimental code). Doing so ensures that pre-registration could not have taken place after data collection [30].

### 5.1. Participants

Forty-six participants took part in the study (ages 20–39, mean = 24.2 ± 4.5; 29 females). Participants gave their informed consent to take part in the experiment. The experiment was approved by the UCL ethics committee (approval numbers 8231/001 and 1260/003). Thirty-five participants met our pre-specified inclusion criteria (ages 20–38, mean = 25.2 ± 4.5; 24 females). We pre-specified a sample size of 35, balancing statistical power and resource considerations. We calculated that with 35 participants, we will have statistical power of 65–85% to replicate our previous findings, assuming no inflation of effect sizes in the original report, and no effect of a reduction in the number of trials per task for group-level sensitivity.

### 5.2. Design and procedure

After a temporally jittered rest period of 500–4000 ms, each trial started with a fixation cross (500 ms), followed by a presentation of a target for 500 ms. In all three conditions, stimuli were dynamic noisy patterns of greyscale values, out of which a grating sometimes emerged and quickly disappeared (figure 1, upper panel). We chose this mode of stimulus presentation in light of indications that it produces stronger metacognitive asymmetries between the perceptions of presence and absence relative to standard static presentation modes [31]. Stimuli consisted of 10 greyscale frames presented at 20 frames per second within a circle of diameter 3°. Stimuli were generated in the following way:

(1) Generate 10 greyscale frames (*F*_1_, …*F*_10_), each an array of 142 by 142 random luminance values.
(2) Create a 142 by 142 sinusudial grating (*G*; 24 pixels per period, random phase). The orientation of the grating is determined according to the trial type.
(3) Determine grating visibility for frame *i* as *p_i_* = *v* × exp(−|*i* − 5|/2) with *v* being the visibility level in this trial (0 for target absent trials).
(4) For each pixel in the frame *F_i_*, *j*, *k*, replace the luminance value for this pixel with the luminance value of this pixel in the grating (*G_j_*, *k*) with a probability of *p_i_*.

Participants performed the following three tasks:

(1) *Discrimination*. Decide whether the grating was tilted clockwise (50% of trials; 45° relative to a vertical baseline) or anticlockwise (−45° relative to a vertical baseline).
(2) *Tilt recognition*. Decide whether the grating was vertical (50% of trials; 0°) or tilted (sampled from a normal distribution with mean 0° and standard deviation *σ*_orientation_). Stimuli were presented with a fixed *v* value of 0.2 at which stimuli are clearly visible.
(3) *Detection*. Decide whether the grating was present (50% of trials) or absent. Gratings in the ‘present’ trials were sampled from a normal distribution with mean 0° and s.d. *σ*_orientation_ (yoked to the tilt-recognition task).

For all three tasks, responses were made with the right-hand index and middle fingers, and response mappings between fingers and stimulus classes were counterbalanced between blocks.

Immediately after making a decision, participants rated their confidence on a six-point scale by using two keys to increase and decrease their reported confidence level with their left-hand thumb, using the same procedure and incentive structure as in Mazor *et al*. [6]. The perceptual decision and the confidence rating phases were restricted to 1000 and 2500 ms, respectively. No feedback was delivered to subjects about their performance.

Participants were acquainted with the task in a preceding behavioural session. During this session, task difficulty was adjusted independently for detection, discrimination and tilt recognition, targeting around 70% accuracy on all three tasks. In detection and discrimination, we achieved this by adaptively controlling the visibility *v* value once in every 10 trials: increasing it when accuracy fell below 60%, and decreasing it when accuracy exceeded 80%. In tilt recognition, *v* was set to 0.20 such that stimuli were highly visible, and calibration was performed on the standard deviation of orientations *σ*_orientation_ in a similar manner. Performance on all three tasks was further calibrated to the scanner environment at the beginning of the scanning session, during the acquisition of anatomical (MP-RAGE and fieldmap) images. After completing the calibration phase, participants underwent five to six 10 min functional scanner runs, each comprising one block of 26 trials from each experimental condition, presented in a random order.

To avoid stimulus-driven fluctuations in confidence, *v* and *σ*_orientation_ were kept fixed within each experimental block. Nevertheless, following experimental blocks with markedly bad (less than or equal to 52.5%) or good (greater than or equal to 85%) accuracy, *v* or *σ*_orientation_ were adjusted for the next block of the same task (divided or multiplied by a factor of 0.95 for bad and good performance, respectively).

### 5.3. Scanning parameters

Scanning took place at the Wellcome Centre for Human Neuroimaging, London, using a 3 Tesla Siemens Prisma MRI scanner with a 64-channel head coil. We used the same sequences as in Mazor *et al*. [6].

### 5.4. Analysis

The pre-registered objectives of this study were to:

1. replicate our finding of an interaction between task (discrimination/detection) and a quadratic effect of confidence on BOLD signal in medial and lateral frontopolar cortex, as well as in the STS and pre-SMA,
2. replicate our finding of an interaction between detection response (present/absent) and the linear effect of confidence on activation in the right TPJ,
3. compare quadratic effects of confidence on activations in the frontopolar cortex, the STS and the pre-SMA in a tilt-recognition task with those in detection and discrimination tasks, and
4. compare response-specific linear effects of confidence on activation in the right TPJ in a tilt-recognition task with those in detection and discrimination tasks.

### 5.5. Exclusion criteria

Individual experimental blocks were excluded in the following cases:

(1) More than 20% of the trials in the block were missed.
(2) Mean accuracy was lower than 60%.
(3) The participant used the same response in more than 80% of the trials.
(4) For a particular response, the same confidence level was reported for more than 90% of the trials.

The first trial of each block was excluded from all analyses, leaving 25 usable trials per block. Subjects were included only if after applying block-wise exclusion specified above, their data had at least three blocks for each task.

### 5.6. fMRI data preprocessing

As in Mazor *et al*. [6], fMRI data preprocessing followed the procedure described in Morales *et al*. [8]. Preprocessing and construction of first- and second-level models used standardized pipelines and scripts available at https://github.com/metacoglab/MetaLabCore. All analyses were carried out using SPM 12 software (https://www.fil.ion.ucl.ac.uk/spm).

### 5.7. Regions of interest

In addition to an exploratory whole-brain analysis (corrected for multiple comparisons at the cluster level), our analysis focused on the following *a priori* regions of interest:

(1) *Medial frontopolar cortex (FPm).* Obtained from a previous connectivity-based parcellation [32].
(2) *Lateral frontopolar cortex (FPl)*. Obtained from [32].
(3) *Brodman area 46 (BA46).* Obtained from [32].
(4) *vmPFC*. Defined as a 8 mm sphere around MNI coordinates [0,46,−7], obtained from a meta-analysis of subjective-value related activations [33] and aligned to the cortical midline.
(5) *rTPJ.* Defined using the contrast confidence_No_ − confidence_Yes_ from Mazor *et al*. [6] (peak voxel [54,−46,26], see mask attached to the protocol folder at ‘ROIs/rTPJ.nii’).
(6) *rSTS*. Defined using the rSTS cluster from the contrast ${\mathrm{confidence}}_{\mathrm{Detection}}^{2}-{\mathrm{confidence}}_{\mathrm{Discrimnation}}^{2}$ from Mazor *et al*. [6] (peak voxel [60,−43,2], see mask attached to the protocol folder at ‘ROIs/rSTS.nii’).
(7) *Pre-supplementary motor area (pre-SMA).* Defined using the pre-SMA cluster from the contrast ${\mathrm{confidence}}_{\mathrm{Detection}}^{2}-{\mathrm{confidence}}_{\mathrm{Discrimnation}}^{2}$ from Mazor *et al*. [6] (peak voxel [0,35,47], see mask attached to the protocol folder at ‘ROIs/preSMA.nii’).

### 5.8. Univariate fMRI analysis

Univariate analysis followed a similar procedure to that described in Mazor *et al*. [6]. After preprocessing, runs were temporally concatenated and a design matrix fitted to the entire time course. Here we chose not to exclude entire runs, but specific blocks of trials. This was achieved by modelling excluded blocks with a separate nuisance regressor. We estimated two sets of design matrices:

### 5.9. Quadratic-confidence design matrix

The quadratic-confidence design matrix (QC-DM) for the univariate GLM analysis consisted of 18 regressors of interest. First, we entered a regressor for each of the six responses: ‘yes’, ‘no’, ‘tilted’, ‘vertical’, ‘clockwise’ and ‘anticlockwise’, modelled by a boxcar regressor with non-zero entries at the 4000 ms interval starting at the onset of the stimulus and ending immediately after the confidence rating phase, convolved with a canonical haemodynamic response function (HRF). Each of these primary regressors was accompanied by two parametric modulators, representing the mean-centred and orthogonalized linear and quadratic effects of confidence.

Trials in which the participant did not respond within the 1000 ms time frame, the first trial of a block, or trials in excluded blocks were modelled by separate regressors. The design matrix also included a run-wise constant term regressor, an instruction-screen regressor for the beginning of each block, motion regressors (the six motion parameters as extracted by SPM in the head motion correction preprocessing phase together with their first derivatives) and regressors for physiological measures (pulse and breathing). Button presses were modelled as stick functions, convolved with the canonical HRF and separated into three regressors: two regressors for each of the two right-hand buttons, and one regressor for both up and down left-hand presses (table 1).

**Table 1.** List of regressors in the QC-DM^2^.

	task	response
1 CW	discrimination	clockwise
2 CW_conf		
3 CW_conf^2^		
4 ACW		anticlockwise
5 ACW_conf		
6 ACW_conf^2^		
7 Y	detection	yes
8 Y_conf		
9 Y_conf^2^		
10 N		no
11 N_conf		
12 N_conf^2^		
13 V	tilt recognition	vertical
14 V_conf		
15 V_conf^2^		
16 T		tilted
17 T_conf		
18 T_conf^2^		

### 5.10. Categorical-confidence design matrices

We also fitted a set of three design matrices—one for each task—in which confidence was modelled as a categorical variable. These design matrices consisted of only one regressor of interest for all included trials, modelled by a boxcar with non-zero entries at the 4000 ms interval starting at the onset of the stimulus and ending immediately after the confidence rating phase, convolved with a canonical HRF. This regressor was in turn modulated by a series of 12 dummy (0/1) parametric modulators—one for every response (‘yes’ and ‘no’ for detection, ‘vertical’ and ‘tilted’ for tilt recognition and ‘clockwise’ and ‘anticlockwise’ for discrimination) and confidence rating (1–6). Using three design matrices instead of one allowed us to set trials from the remaining two tasks to serve as a baseline for the task of interest. These design matrices included the same set of nuisance regressors as the main design matrix.

For each participant, beta-estimates from the categorical-confidence design matrices were used as input to six response-specific multiple linear regression models, with linear confidence and quadratic-confidence terms as predictors, in addition to an intercept term. Subject-specific coefficients were then subjected to ordinary least-squares (OLS) group-level inference, to estimate linear and quadratic effects of confidence on univariate brain activation and compare these effects between responses. The rationale for employing this two-step approach is its indifference to differences in the confidence distributions for the six responses, which may bias the estimation of quadratic and linear terms. Furthermore, linear and quadratic regressors were not orthogonalized, and instead competed to explain variance in the data, minimizing interpretational ambiguity that may arise when using default orthogonalization settings [34].

### 5.11. Representational similarity analysis

RSA [19] was used to detect consistent spatio-temporal structures in the representation of choice and confidence across tasks and responses, within our seven pre-specified ROIs. High- and low-confidence trials were defined using a median split within each response category. The empirical RDM was then compared against the following set of *a priori* theoretical RDMs:

(1) *Task* (figure 4*a*). Trials of the same task are similar; trials of different tasks are different.
(2) *Variance structure* (figure 4*b*). Discrimination trials are similar to each other. Detection ‘present’ trials and tilt-recognition ‘tilted’ trials are similar (high variance), and detection ‘absent’ trials are similar to tilt-recognition ‘vertical’ trials (low variance).
(3) *Decision: detection only* (figure 4*c*). Detection ‘yes’ responses are different from detection ‘no’ responses, with no consistent differences between tilt-recognition or discrimination responses.
(4) *Decision: unequal variance only* (figure 2*d*). Detection ‘yes’ responses are different from detection ‘no’ responses, and ‘tilted’ responses are different from ‘vertical’ responses in the tilt-recognition task, with no consistent differences between the two discrimination responses.
(5) *Confidence* (figure 4*e*). High- and low-confidence trials are represented differently, without an effect of task or response.
(6) *Confidence and variance structure interaction* (figure 4*f*). High- and low-confidence trials are represented differently. This effect is modulated by the variance structure of the trial category.
(7) *Confidence in detection only* (figure 4*g*). High- and low-confidence trials are represented differently in detection only.
(8) *Confidence in unequal variance only* (figure 4*h*). High- and low-confidence trials are represented differently in detection and tilt recognition only.

Since tasks were presented in different blocks, a high degree of similarity between conditions within a task could emerge due to temporal autocorrelations in physiological and physical noise, irrespective of distances in neural representations. To control for this, neural RDMs were constructed from distances between pairs of conditions from distinct experimental runs, and never within a single run. We chose Euclidean distance as our dissimilarity measure in order to be sensitive to differences in overall activity between tasks and responses, in addition to the relative activation patterns of voxels within an ROI.

For each ROI, the lower bound of the noise ceiling was defined as the average Spearman correlation between a given participants' empirical RDM and the average ranked RDM of all other participants [21]. This number reflects the shared variance between the RDMs of different participants that can be captured by any theoretical RDM.

### 5.12. Group-level inference

For exploratory whole-brain analysis, group-level inference followed an OLS procedure on the subject-specific contrast maps. Correction for multiple comparisons was performed at the cluster level, using a significance threshold of *p* = 0.05 and a cluster defining threshold of *p* = 0.001. No correction for multiple comparisons was applied to our pre-specified ROIs.

Bayes factors were extracted by following the method described in Rouder *et al*. [18]. Whenever an expected effect size could be estimated from Exp. 1, we used it as a scaling factor for the prior distribution over effect sizes, reflecting a belief that if an effect exists, there is a probability of 0.5 that it is weaker than what we had observed in Exp. 1. Whenever an effect size could not be reliably estimated, we used the default scaling factor of $\sqrt{2}/2$ .

## Data Availability

All raw imaging data is freely available on OpenNeuro: https://openneuro.org/datasets/ds004081/versions/1.0.0. Full analysis scripts and summary statistics from regions of interest are available on GitHub: https://github.com/matanmazor/unequalVarianceDiscrimination and have been archived within the Zenodo repository: https://doi.org/10.5281/zenodo.7351965 [35]. The data are provided in the electronic supplementary material [36].

## Footnotes

1 We thank Heleen Slagter for suggesting this idea.

2 Due to a mistake, our pre-registration document specified that trials will be classified based on the presented stimulus rather than the response. This would mean that high confidence hits would be classified together with high-confidence misses, as in both cases the stimulus was a grating. We therefore decided to follow the analysis strategy of Mazor *et al.* [6] and classify trials based on response instead.

## References

[1] Mamassian P. 2016 Visual confidence. Annu. Rev. Vision Sci. **2**, 459-481. 10.1146/annurev-vision-111815-114630 28532359

[2] Vaccaro AG, Fleming SM. 2018 Thinking about thinking: a coordinate-based meta-analysis of neuroimaging studies of metacognitive judgements. Brain Neurosci. Adv. **2**, 2398212818810591. (10.1177/2398212818810591) 30542659 PMC6238228

[3] Treisman M, Williams TC. 1984 A theory of criterion setting with an application to sequential dependencies. Psychol. Rev. **91**, 68.

[4] Ko Y, Lau H. 2012 A detection theoretic explanation of blindsight suggests a link between conscious perception and metacognition. Phil. Trans. R. Soc. B **367**, 1401-1411. 22492756 10.1098/rstb.2011.0380 PMC3318762

[5] Fleming SM. 2020 Awareness as inference in a higher-order state space. Neurosci. Consciousness **2020**, niz020. 10.1093/nc/niz020 PMC7065713 32190350

[6] Mazor M, Friston KJ, Fleming SM. 2020 Distinct neural contributions to metacognition for detecting, but not discriminating visual stimuli. ELife **9**, e53900. (10.7554/eLife.53900) 32310086 PMC7170652

[7] Bang D, Fleming SM. 2018 Distinct encoding of decision confidence in human medial prefrontal cortex. Proc. Natl Acad. Sci. USA **115**, 6082-6087. (10.1073/pnas.1800795115) 29784814 PMC6003322

[8] Morales J, Lau H, Fleming SM. 2018 Domain-general and domain-specific patterns of activity supporting metacognition in human prefrontal cortex. J. Neurosci. **38**, 3534-3546. (10.1523/JNEUROSCI.2360-17.2018) 29519851 PMC5895040

[9] Wickens TD. 2001 Elementary signal detection theory. Oxford, UK: Oxford University Press.

[10] Kellij S, Fahrenfort J, Lau H, Peters MAK, Odegaard B. 2021 An investigation of how relative precision of target encoding influences metacognitive performance. Attent. Percept. Psychophys. **83**, 512-524. (10.3758/s13414-020-02190-0) PMC7875845 33244733

[11] Mazor M, Dijkstra N, Fleming SM. 2022 Dissociating the neural correlates of subjective visibility from those of decision confidence. J. Neurosci. **42**, 2562-2569. 35121637 10.1523/JNEUROSCI.1220-21.2022 PMC8944226

[12] Mazor M. 2021 Inference about absence as a window into the mental self-model. *PsyArXiv*. (10.31234/osf.io/zgf6s)

[13] Denison RN, Adler WT, Carrasco M, Ma WJ. 2018 Humans incorporate attention-dependent uncertainty into perceptual decisions and confidence. Proc. Natl Acad. Sci. USA **115**, 11 090-11 095. (10.1073/pnas.1717720115) PMC6205425 30297430

[14] Rahnev D. 2021 Response bias reflects individual differences in sensory encoding. Psychol. Sci. **32**, 1157-1168. (10.1177/0956797621994214) 34197259 PMC8641135

[15] Mazor M, Moran R, Fleming SM. 2021 Metacognitive asymmetries in visual perception. Neurosci. Conscious. **2021**, niab025. (10.1093/nc/niab025) 34676104 PMC8524176

[16] Meuwese JDI, van Loon AM, Lamme VAF, Fahrenfort JJ. 2014 The subjective experience of object recognition: comparing metacognition for object detection and object categorization. Attent. Percept. Psychophys. **76**, 1057-1068. (10.3758/s13414-014-0643-1) 24554231

[17] Aberbach-Goodman S, Buaron B, Mudrik L, Mukamel R. 2021 Same action, different meaning: neural substrates of semantic goal representation. Cereb. Cortex **32**, 4293-4303. (10.1093/cercor/bhab483) 35024783

[18] Rouder JN, Speckman PL, Sun D, Morey RD, Iverson G. 2009 Bayesian *t* tests for accepting and rejecting the null hypothesis. Psychon. Bull. Rev. **16**, 225-237. (10.3758/PBR.16.2.225) 19293088

[19] Kriegeskorte N, Mur M, Bandettini P. 2008 Representational similarity analysis—connecting the branches of systems neuroscience. Front. Syst. Neurosci. **2**, 4. (10.3389/neuro.06.004.2008) 19104670 PMC2605405

[20] Ritchie JB, Bracci S, de Beeck HO. 2017 Avoiding illusory effects in representational similarity analysis: what (not) to do with the diagonal. Neuroimage **148**, 197-200. 28069538 10.1016/j.neuroimage.2016.12.079

[21] Nili H, Wingfield C, Walther A, Su L, Marslen-Wilson W, Kriegeskorte N. 2014 A toolbox for representational similarity analysis. PLoS Comput. Biol. **10**, e1003553. (10.1371/journal.pcbi.1003553) 24743308 PMC3990488

[22] Graziano MS, Webb TW. 2015 The attention schema theory: a mechanistic account of subjective awareness. Front. Psychol. **6**, 500. 25954242 10.3389/fpsyg.2015.00500 PMC4407481

[23] Cremers HR, Wager TD, Yarkoni T. 2017 The relation between statistical power and inference in fMRI. PLoS ONE **12**, e0184923. 29155843 10.1371/journal.pone.0184923 PMC5695788

[24] Christensen MS, Ramsøy TZ, Lund TE, Madsen KH, Rowe JB. 2006 An fMRI study of the neural correlates of graded visual perception. Neuroimage **31**, 1711-1725. (10.1016/j.neuroimage.2006.02.023) 16626975

[25] De Martino B, Bobadilla-Suarez S, Nouguchi T, Sharot T, Love BC. 2017 Social information is integrated into value and confidence judgments according to its reliability. J. Neurosci. **37**, 6066-6074. (10.1523/JNEUROSCI.3880-16.2017) 28566360 PMC5481942

[26] Lebreton M, Abitbol R, Daunizeau J, Pessiglione M. 2015 Automatic integration of confidence in the brain valuation signal. Nat. Neurosci. **18**, 1159-1167. 26192748 10.1038/nn.4064

[27] Davidson MJ, Macdonald JSP, Yeung N. 2021 Alpha power and stimulus-evoked activity dissociate metacognitive reports of attention, visibility and confidence in a visual detection task. *bioRxiv*. (10.1101/2021.11.23.469669)

[28] Goldman RI, Stern JM, Engel J, Cohen MS. 2002 Simultaneous EEG and fMRI of the alpha rhythm. Neuroreport **13**, 2487-2492. (10.1097/01.wnr.0000047685.08940.d0) 12499854 PMC3351136

[29] Laufs H, Kleinschmidt A, Beyerle A, Eger E, Salek-Haddadi A, Preibisch C, Krakow K. 2003 EEG-correlated fMRI of human alpha activity. Neuroimage **19**, 1463-1476. 12948703 10.1016/s1053-8119(03)00286-6

[30] Mazor M, Mazor N, Mukamel R. 2019 A novel tool for time-locking study plans to results. Europ. J. Neurosci. **49**, 1149-1156. (10.1111/ejn.14278) 30462871

[31] Maniscalco B, Lau H. 2011 On a distinction between detection and discrimination: metacognitive advantage for signal over noise. J. Vis. **11**, 163.

[32] Neubert FX, Mars RB, Thomas AG, Sallet J, Rushworth MFS. 2014 Comparison of human ventral frontal cortex areas for cognitive control and language with areas in monkey frontal cortex. Neuron **81**, 700-713. (10.1016/j.neuron.2013.11.012) 24485097

[33] Bartra O, McGuire JT, Kable JW. 2013 The valuation system: a coordinate-based meta-analysis of BOLD fMRI experiments examining neural correlates of subjective value. Neuroimage **76**, 412-427. (10.1016/j.neuroimage.2013.02.063) 23507394 PMC3756836

[34] Mumford JA, Poline JB, Poldrack RA. 2015 Orthogonalization of regressors in fMRI models. PLoS ONE **10**, e0126255. (10.1371/journal.pone.0126255) 25919488 PMC4412813

[35] Mazor M, Gong C, Fleming SM. 2023 Code for: Re-evaluating frontopolar and temporoparietal contributions to detection and discrimination confidence. Zenodo. (10.5281/zenodo.7351965) PMC10113806 37090969

[36] Mazor M, Gong C, Fleming SM. 2023 Re-evaluating frontopolar and temporoparietal contributions to detection and discrimination confidence. Figshare. (10.6084/m9.figshare.c.6605118) PMC10113806 37090969

